# IKKe in osteoclast inhibits the progression of methylprednisolone-induced osteonecrosis

**DOI:** 10.7150/ijbs.57962

**Published:** 2021-03-30

**Authors:** Yingjie Liu, Haojie Shan, Yang Zong, Yiwei Lin, Wenyang Xia, Nan Wang, Lihui Zhou, Youshui Gao, Xin Ma, Chaolai Jiang, Xiaowei Yu

**Affiliations:** 1 Department of Orthopaedic Surgery, Shanghai Jiao Tong University Affiliated Sixth People's Hospital, Shanghai 200233, China.; 2 Department of Emergency, the First Affiliated Hospital of Zhengzhou University, Zhengzhou, 450052, Henan, China.; 3 Department of Orthopaedic Surgery, Xiangshan First People's Hospital, Ningbo 315700, Zhejiang, China.

**Keywords:** ONFH, IKKe, non-canonical NF-κB pathway, osteoclasts.

## Abstract

Previous studies have described that NF-κB signaling mediated by NFκB-inducing kinase (NIK) plays a critical role of the differentiation of osteoclasts. We aim to explore the role of IKKe in methylprednisolone -induced osteonecrosis of the femoral head (ONFH). Methylprednisolone-induced ONFH mice model was successfully established, and subjected to micro computed tomography to detect the femoral head image of the mice. Bone marrow cells from experimental mice were collected and cultured. qPCR and immunoblot were performed to examine the possible signal pathways of IKKe involvement, and osteoclast-related gene expressions in IKKe^+/+^ and IKKe^-/-^ cells *in vitro* and *in vivo* were examined. It was found that the levels of IKKe decreased in ONFH patients, and IKKe interacted with NIK in the NF-κB signal pathway to suppress osteoclasts *via* inhibiting the transcription of NIK. Furthermore, IKKe knockout promoted the osteoclastogenesis in mice model. Finally, IKKe knockout suppressed methylprednisolone-induced ONFH and pro-inflammatory responses in mice model. Our findings show a mechanism of IKKe inhibition of the progression of methylprednisolone-induced ONFH via the NIK/NF-κB pathway.

## Introduction

Osteonecrosis of the femoral head (ONFH), characterized by the apoptosis of osteoblasts and osteocytes, and the activation of osteoclasts, results from a compromise of blood supply to the femoral head 1. Femoral head necrosis inevitably causes pain, joint movement and weight-bearing walking dysfunction. The insufficient blood supply of the area results in osteocytes necrosis. The changes in the structure of the femoral head, collapse and deformation of the femoral head can be observed in radiography images 2. The main side effects of glucocorticoid (GC) are osteonecrosis and fracture. GC-induced osteonecrosis of the femoral head occurs in 9-40% of patients when they accept administration of long-term glucocorticoid therapy 3. Studies have proved that excessive dose of glucocorticoid decreases osteoclastogenesis, increases the apoptosis of osteocytes and the survival of osteoclast, which subsequently breaks the balance of bone remodeling and promotes bone resorption 4.

The current known cause of osteonecrosis is the activation of osteoclasts and inhibition of osteocytes, which results in an imbalance of bone remodeling 5. Previous studies have described that RANKL in the NF-κB signal pathway enables the enhancement of the viability of mature osteoclasts and prevents osteoclast apoptosis, and non-canonical NF-κB signaling mediated by NF-κB-inducing kinase (NIK) plays a critical role of osteoclast differentiation in immune cells 6-9. We found that osteoclasts in the inflammation site of patients with femoral head necrosis have very low expression of the inhibitor of nuclear factor kappa-B kinase epsilon (IKKe). IKKe was reported to participate in immune response, promote cell proliferation/survival and join insulin signaling 10. However the role of IKKe in osteoclasts has not fully been understood, what component IKKe regulating and what signaling pathway it involved in the occurrence of femoral head necrosis, and the molecular mechanism that promotes/suppresses the formation of osteoclasts, have not yet been identified. In this study, we hypothesize that IKKe can be used as an inhibitor of NIK. Low expression of IKKe will cause the accumulation of NIK, which indirectly leads to an increase of osteoclasts, which intensifies the severity of femoral head necrosis.

## Materials and methods

### Animals

Wild-type (WT) mice on C57BL/6 background at 8 weeks old and IKKe knockout mice with B6 background were purchased from Animal Model Research Center of Nanjing University (Nanjing, China). The animals were kept under controlled condition, which was the temperature at 22-24°C and humidity at 60±5%. These mice were bred and mated in a specific pathogen-free facility. PCR analysis was applied for genotyping, only the IKKe^+/+^ (WT) and IKKe^-/-^ mice were selected for further experiments. Animal studies were approved by Shanghai Jiao Tong University Affiliated Sixth People's Hospital.

### Cell culture

Bone marrow cells were collected from the tibia and femurs of age-matched mice (6-8 weeks), and sorted with CD11b+ antibody (Abcam, Cambridge, MA) by flow cytometer. After cultured in DMEM supplemented with M-CSF-containing conditioned medium (30%) and fetal bovine serum (20%, Invitrogen, Waltham, MA) for 4-6 days, the marrow-derived macrophages were considered to be ready for further experiments 9. For the osteoclast differentiation, M-CSF-conditioned media was added to the bone marrow-derived macrophages for 3 days, with adding the receptor activator of nuclear factor kappa-Β ligand (RANKL, 188-02291, Wako, Tokyo, Japan) at 25-100 ng/ml and cultured the cells for another 3-4 days. Lipopolysaccharides (LPS, L2880, Sigma-Aldrich, St. Louis, MO) was used for further experiments.

### Established ONFH animal model

Sixty healthy female mice (8-week-old) were used in this study. There were 10 mice in each group. To induce ONFH in the mice, methylprednisolone (MPS, Pfizer, Shanghai, China) (20 mg/kg/d) was intramuscularly injected into the mice. On the first three weeks, MPS was injected on the day 1 to day 3 and rested on day 4 to day 7, then the mice were rested for another three weeks. The administration schedule was total six weeks. After anesthetized, the femoral heads of the mice were assessed by micro-CT, immunohistochemically and histological analyses 11, 12. All the experimental mice were alive and no antibiotics were administrated by the end of dispose.

### Micro-CT analysis

The femoral heads in each group were carefully separated with surgical knife, following soaked in 10% formalin solution for overnight. These specimens were analyzed by Micro-CT (SkyScan1178, Kontich, Belgium). The representative specimens of each group were set at a 9 µm per pixel in their sagittal planes, the images were automatically generated by the DataViewer software of the machine.

### RNA sequence analysis (RNAseq)

CD11b+ cells were collected from peripheral blood of ONFH patients (three cases) and healthy donors (three cases). One µg of total RNAs were extracted using RNeasy kit (Cat. No. 80404, Qiagen, Valencia, CA). The quality of RNAs were examined by bioanalyzer, an RNA Integrity Number (RIN)>9.8 was accepted. For performing RNAseq, the RNA library was made by Total RNA-Seq Library Prep Kit (Cat. No. 095.24, Lexogen, Vienna, Austria). The next generation sequencing was performed by Novogene (Beijing, China), and data were also analyzed by Novogene.

### Immunoblots

Antibodies for NIK (H248, sc-8417) and IKKα (H-744, sc-7606) were from Santa Cruz Biotechnology (Dallas, TX). Antibody for Actin (C-4, A2066) and mouse phospho-IKKe (06-1340) were from Sigma. Antibodies for human phospho-IKKe (#8766), IKKe (#3416) and mouse phospho-IKKα/β (#2697) were purchased from Cell Signaling Technology (Danvers, MA).

For Western blot experiments, the cells were lysed on ice in 1× lysis buffer (Cat. No.00-4333-57, Thermo, Waltham, MA) with 1× Protease Inhibitor Cocktail (Cat no. 78429, Thermo) or 1× phosphatase inhibitor cocktail set II (Calbiochem, La Jolla, CA). Protein expression was measured by Micro BCA Protein Assay Kit (#23235, Thermo). Ten micrograms of total protein from each sample were loaded on 10% SDS Bis-Tris.Cl polyacrylamide gel with running buffer at 100V voltage for 1 hour, and transferred onto 0.2 μm PVDF membranes (Bio-Rad, Hercules, CA) at 100mA for overnight at 4°C. The membranes were incubated in 5% dry mike on shaker for 1 hour, followed by probed with various first antibodies of 2 hours and incubated with corresponding second antibody for 1 hour. Images were achieved by using an ECL system (Super Signal west Dura Kit, Thermo).

### Quantitative reverse transcription PCR (qRT-PCR)

Total RNA was extracted from bone marrow cells using RNA extraction mini kit (QIAGEN). cDNA synthesis was carried out using iScript Reverse Transcription Supermix (#170-8841, Bio-Rad). Primer sequences were presented in the supplementary materials.

Quantitative RT-PCR was performed using the SsoAdvanced Universal SYBR Green Supermix (#172-5124, Bio-Rad) and run on standard cycle conditions followed the manufacturer's protocol.

### Enzyme-linked immunosorbent assay (ELISA)

The cells were cultured in a 96-well plate. 50 µL of supernatant was added to each well of a 96-well plate, which the antibody pre-coated with (R&D systems Inc. Minneapolis, USA) 13. 50 µL of propotional diluted Standards were also added into the wells with duplicate. Plate was incubated at room tempreture for 1 h then washed out 3 times. Next, working substrate solution (100 µL) was added to the wells, incubated for 20 minutes following which 50 µL of Stop Solution to quench the reaction. The values were measured by a plate reader and the values were calculated by standard curve from prepared standard solutions.

### Statistics

Data are mean ± SEM, and analyzed by SPSS 13.0 (SPSS, Chicago, IL). Statistics were examined by Student's t test or one-way ANOVA analysis with a Tukey's post hoc test. Statistical significance was set when p value was less than 0.05.

## Results

### The expression of IKKe was reduced in ONFH patients

**Figure 1:**
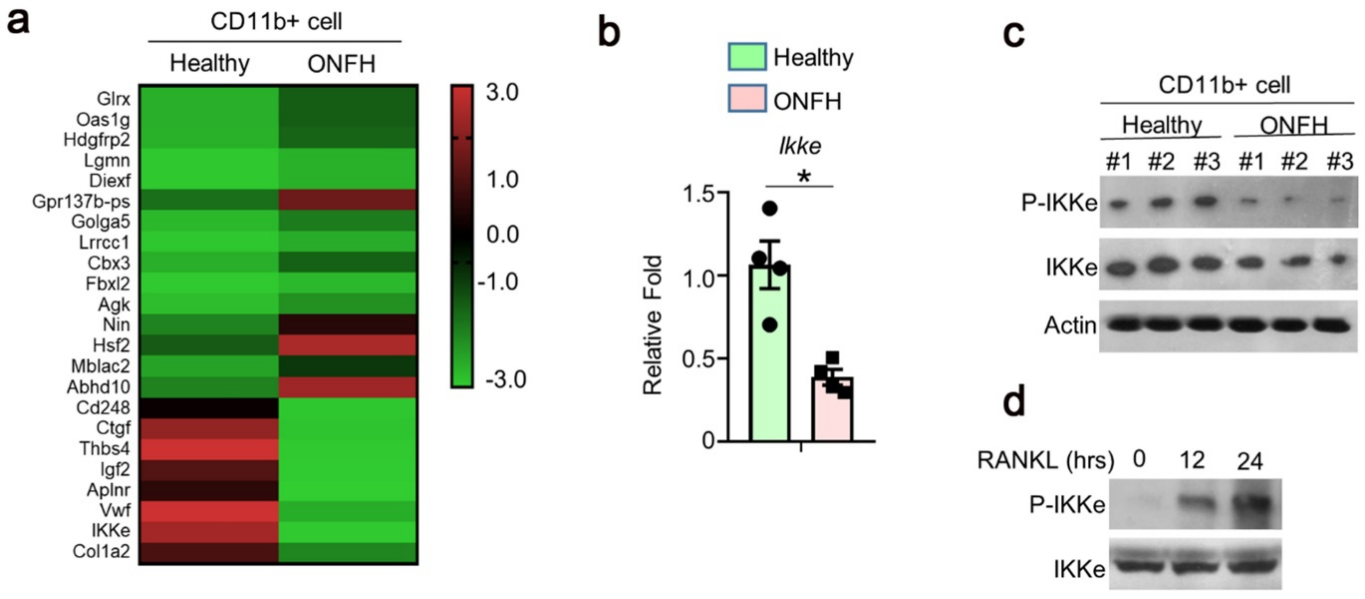
** The expression of IKKe is reduced in ONFH patients.** (a) The CD11b+ cells in PBMCs from healthy donors and ONFH patients were isolated by FACS. The differences of multiple gene expressions in were analyzed by RNAseq. (b-c) The mRNA (b) and protein level of IKKe in CD11b+ cells in PBMCs from healthy donors and ONFH patients were measured by qPCR (n=4) and immunoblot (n=3) (c). (d) Cells were cultured in RANKL (50 ng/ml) and M-CSF for indicated days. The phosphorylation of IKKe induced by RANKL was evaluated by IB at indicated time point. Relative mRNA folds for each gene were calculated based on the Actin by Bio-Rad software. Data are mean ± SEM. Representative experiments were performed at least three independent times. *P < 0.05.

To investigate the genes involved in the regulation of osteoclasts, 3 cases of ONFH patients and 3 cases of health donors were selected. We performed RNA sequence (RNAseq) using CD11B+ (Pan-myeloid) cells separated from peripheral blood (peripheral blood mononuclear cells, PBMCs). The differences of multiple gene expressions were analyzed using RNAseq, which showed great expression differences (Fig. 1a). We performed qPCR and found the expression of IKKe was one-fold lower in ONFH patients than health donors (Fig. 1b). In the results of immunoblot, the expression of IKKe and phosphorylation of IKKe (P-IKKe) were both reduced compared with healthy donors (Fig. 1c). Then we collected bone marrow cells from the healthy donors and those cells were cultured. The P-IKKe induced by RANKL was present at 12 hours and increased at 24 hours by the performance of immunoblot (Fig. 1d). These results indicated that the expression of IKKe was reduced in ONFH patients, moreover, the osteoclastogenesis inducer RANKL could phosphorylate IKKe.

### Antiviral signal pathway was not involved in RANKL-induced activation of IKKe

**Figure 2:**
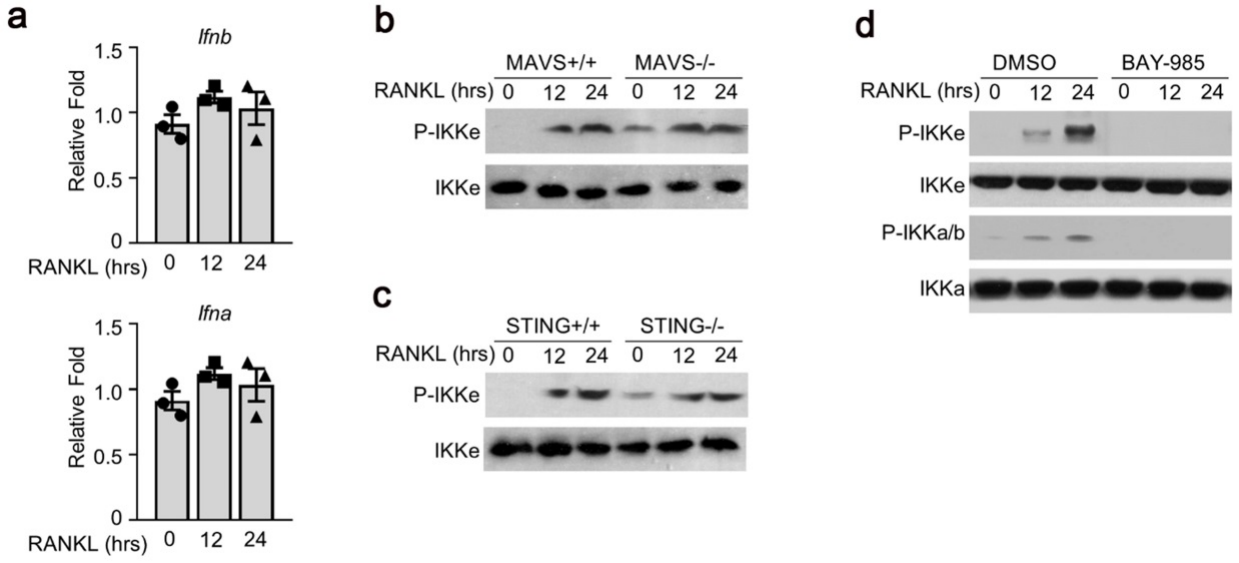
** Antiviral signal pathway was not involved in RANKL-induced activation of IKKe.** (a) Cells were cultured in RANKL (50 ng/ml) and M-CSF for different time points. The expressions of Ifna and Ifnb were determined by qPCR (n=3). (b-c) WT, MAVS-/- or STING-/- cells were cultured in M-CSF for 3 days and then added RANKL (50 ng/ml) for indicated time point. The phosphorylation of IKKe induced by RANKL was evaluated by IB. (d) WT cells were cultured in M-CSF for 3 days and then added RANKL (50 ng/ml) together with IKKs inhibitor BAY-985 (2 nM) for indicated time point. The phosphorylation of IKKb and IKKe induced by RANKL was evaluated by IB. Relative mRNA folds for each gene were calculated based on the Actin by Bio-Rad software. Data are mean ± SEM. Representative experiments were performed at least three independent times.

To examine the possible mechanism(s) of RANKL-induced IKKe phosphorylation, we tested several signal pathways related to IKKe activation. First, we examined interferon a/b pathway. We were not able to detect any obvious differences in the expressions of interferon a and b (Fig. 2a). Next, we used the bone marrow cells from mitochondrial antiviral-signaling protein (MAVS) or stimulator of interferon genes (STING) mice. The wild type (MAVS^+/+^ or STING^+/+^) or knockout type (MAVS^-/-^ or STING^-/-^) bone marrow cells were cultured in M-CSF for 3 days which were then added with RANKL (50 ng/ml) for 12 and 24 hours. The phosphorylation of IKKe induced by RANKL was evaluated by immunoblot. The result showed the P-IKKe was both induced by RANKL in MAVS^+/+^ or MAVS^-/-^, no obvious differences were observed. Bone marrow cells from STING mice presented a similar result (Fig. 2b, 2c). Finally, we found an IKKα/β inhibitor, BAY-985, could inhibit the phosphorylation of IKKe, suggesting that BAY-985 might act as the kinase of IKKe (Fig. 2d). These data demonstrated that antiviral signal pathways were not involved in RANKL-induced IKKe activation.

### IKKe negatively regulate the protein level of NIK

**Figure 3:**
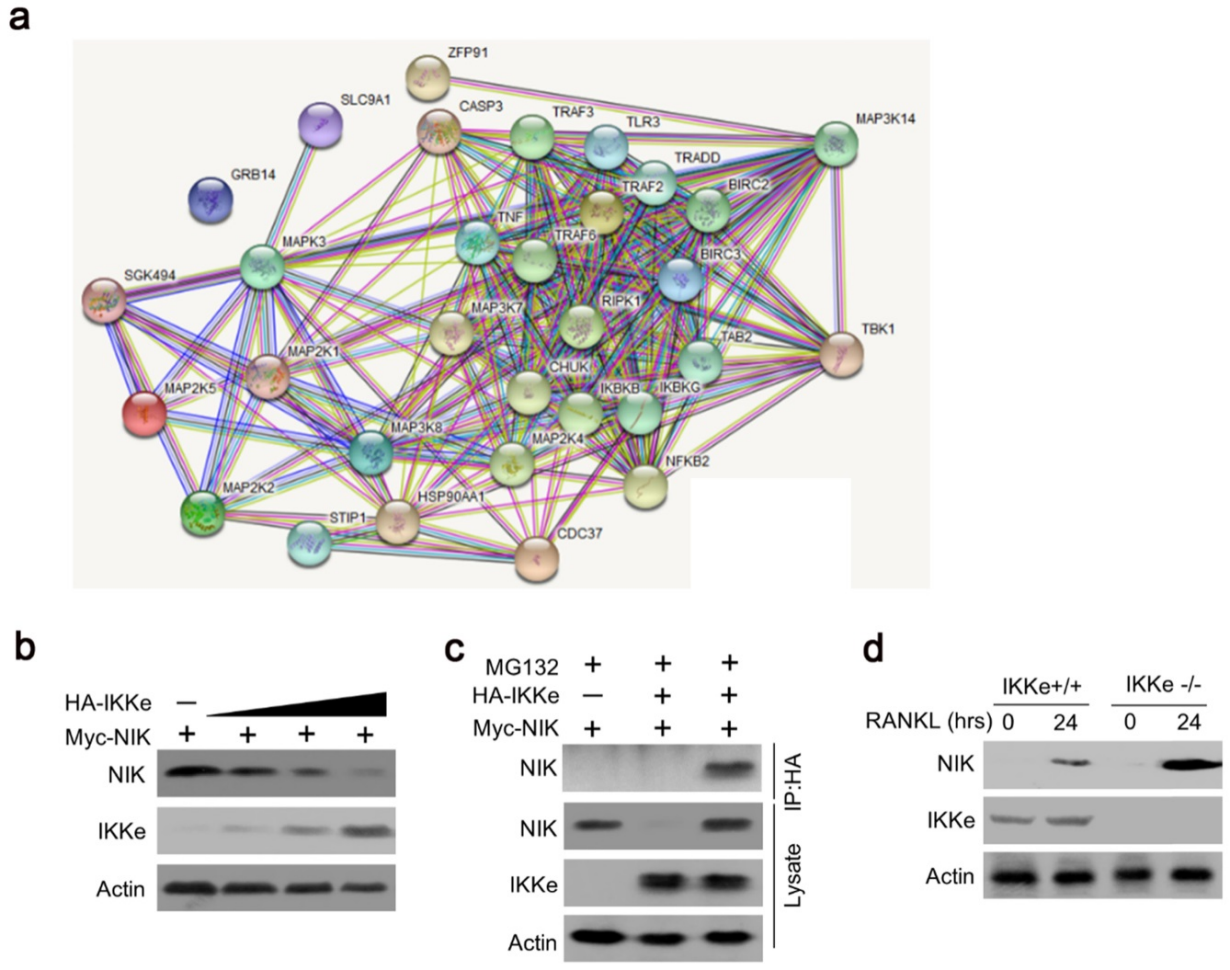
** IKKe negatively regulate the protein level of NIK.** (a) The interacted proteins of NIK were presented in STRING Interaction Network. (b) HEK293 cells were transfected with various combinations of expression vectors for 48 h, then the whole cell lysates were subjected to Western blot analysis. (c) Immunoassay of HEK293T cells transfected with various combinations of expression vectors for HA-tagged IKKe (HA-IKKe) and Myc-tagged NIK (HA-NIK) followed by immunoprecipitation of proteins from lysates. (d) Immunoblot analysis of NIK and actin in whole-cell lysates of wild-type and IKKe KO osteoclasts stimulated with RANKL (50 ng/ml). Data are mean ± SEM and representative of at least three independent experiments.

To further elucidate the IKKe regulation for osteoclasts in NF-κB signal pathway, we searched the related genes which IKKe interacted with via STRING Interaction Network. Results showed that TBK1, one of the factors of IKKe family interacted with NIK (MAP3K14) via non-canonical pattern in the NF- κB pathway (Fig. 3a). Hence, we suggest that IKKe regulate NIK to stimulate osteoclast formation. To prove this hypothesis, we performed immunoblot analysis in HEK293 cells when co-transfected with HA-IKKe and Myc-NIK for 48 hours with gradient increasing of HA-IKKe expression vectors. Our data presented that, in with the increase in HA-IKKe expression vectors, the expression of NIK decreased. Meanwhile, the expression of IKKe increased, which indicate that IKKe interacts with NIK and suppresses its expression (Fig. 3b). To further investigate whether IKKe directly interacted with NIK, we performed immunoprecipitation. Vectors carrying various tag HA or Myc co-transfected to HEK293T cells with the empty vector MG132, three types of transfected cells were collected: MG132+, HA-IKKe-, Myc-NIK+; MG132+, HA-IKKe+, Myc-NIK-; and MG132+, HA-IKKe+, Myc-NIK+. Immunoprecipitation was performed with the HA-tag antibody followed by immunoblot with NIK. Whole cell lysates were also examined with NIK, IKKe and Actin antibodies. The results clearly showed when IP with HA-tag antibody, NIK antibody was unable to be detected any signals when lacking of either of the NIK or IKKe, while when both IKKe and NIK existed, NK detected the signal, which meant IKKe interacted directly with NIK. The whole lysate blotted with NIK, IKKe and actin further proved our hypothesis (Fig. 3c). Immunoprecipitation of proteins from lysates with anti-HA and immunoblot analysis with Myc were done after transfection with different combo of vectors expressing Myc-tagged NIK (HA-NIK) and HA-tagged IKKe (HA-IKKe). Then we knocked out IKKe in osteoclasts, in the stimulation with RANKL, the expression of NIK was much stronger than wild-type osteoclasts (Fig. 3d). These results indicate that IKKe directly interacted with NIK and negatively regulated it.

### IKKe deficiency promoted osteoclastogenesis and expression of osteoclast-related genes

**Figure 4:**
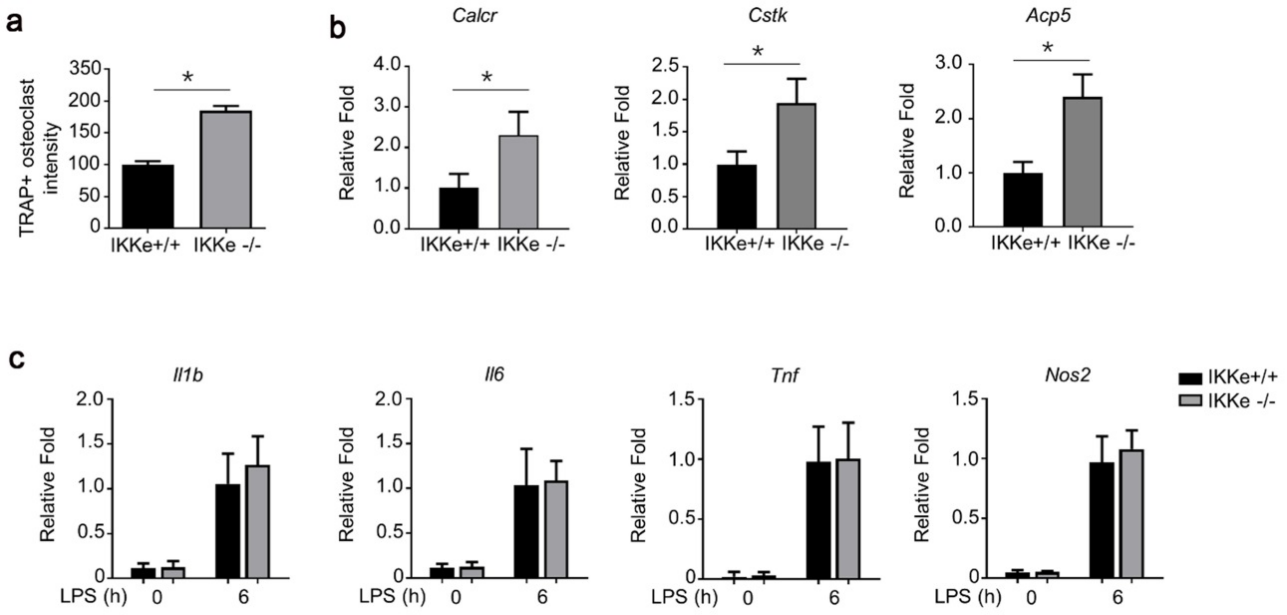
** IKKe deficiency promoted osteoclastogenesis and expression of osteoclast-related genes.** (a) TRAP staining was employed to analyze osteoclast differentiation from bone marrow cells. TRAP activity was determined by an ELISA reader at 410 nm (optical density). (b) Expression of osteoclast genes, including Cstk, Acp5 and Calcr, were determined by qPCR in the cells cultured for 3 days in RANKL (50 ng/ml) and M-CSF. (c) The levels of cytokines in WT and IKKe-/- osteoclast treated with LPS (100 ng/ml) were analyzed by qRT-PCR (n=4). Relative mRNA folds for each gene were calculated based on the Actin by Bio-Rad software. Data are mean ± SEM. Representative experiments were performed at least three independent times. *P < 0.05.

To test the regulation of IKKe in osteoclasts, we cultured wild type of IKKe (IKKe^+/+^) and IKKe knock out (IKKe^-/-^) bone marrow cells from corresponding mice. The TRAP activity in IKKe^-/-^ was one-fold higher than that of IKKe^+/+^ (Fig. 4a). We also examined the osteoclast-related genes in IKKe^+/+^ and IKKe^-/-^ bone marrow cells (Cstk, Acp5 and Calcr) when these cells were cultured in M-CSF and RANKL (50 ng/ml) for 3 days. RNA levels of osteoclast-related genes by qPCR all achieved higher folds compared with wild type, and the differences were obvious (Fig. 4b). We also checked proinflammatory cytokines in WT and IKKe^-/-^ osteoclast treated with LPS (100 ng/ml) by qRT-PCR. Results showed no obvious differences of the expressions of II1b, II6, Tnf and Nos2 in WT and IKKe^-/-^ (Fig. 4c). These experiments showed IKKe knocked out bone marrow cells promoted the osteoclastogenesis and increased the RNA levels of osteoclast-related genes.

### IKKe presented bone tissue-protective effects in methylprednisolone-treated mice

**Figure 5:**
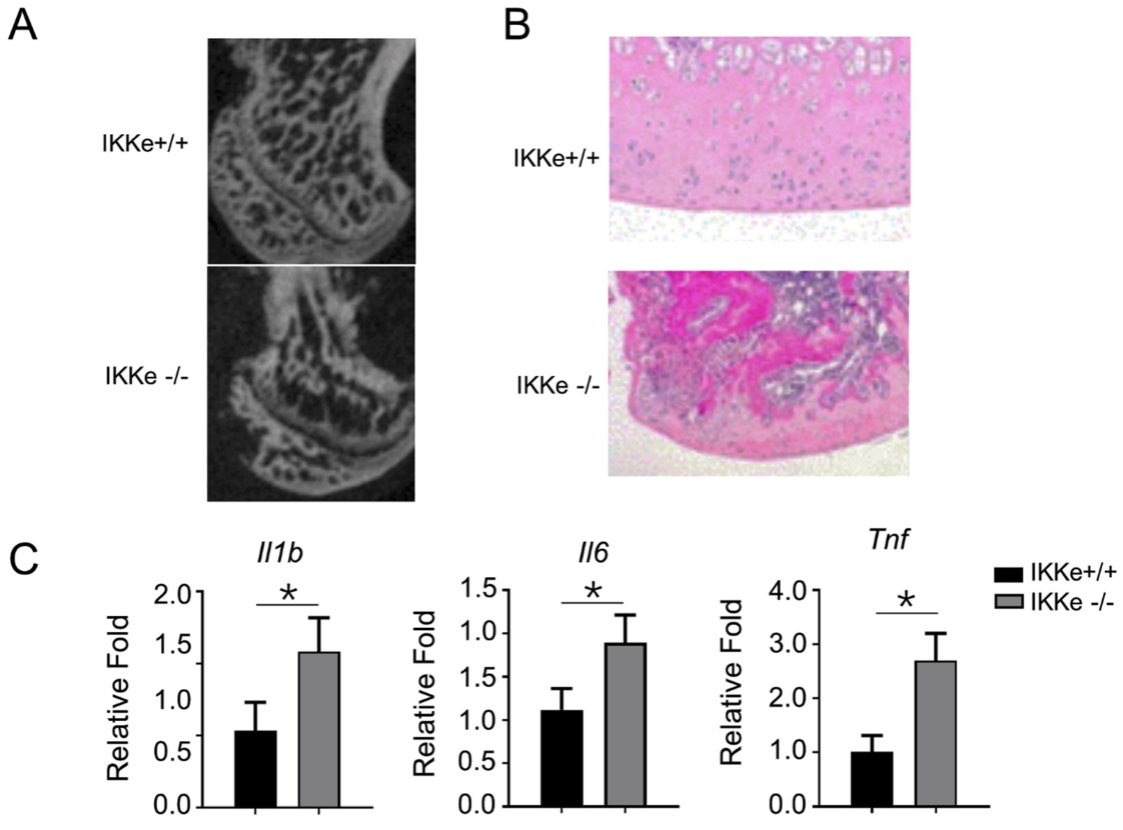
** IKKe presented protective effects in bone tissues in methylprednisolone-treated mice.** (a) Sagittal images of the bones within the femoral heads in MPS-treated WT and IKKe-/- mice. (b) H&E images of the femoral heads in WT and IKKe-/- mice treated with MPS. Scale bar: 100 μm. (c) mRNA levels in bone tissue from WT and IKKe-/- mice with ONFH were examined using qRT-PCR (n=4). All data are presented as folds were calculated based on the Actin by Bio-Rad software. Data are mean ± SEM. Representative experiments were performed at least three independent times. *P < 0.05.

Finally, we examined the osteoclastogenesis and the pro-inflammatory expressions in IKKe^+/+^ and IKKe^-/-^ methylprednisolone (MPS)-induced ONFH mice model (Fig. 5). In Fig. 5a, the micro-CT images clearly showed bone structure collapse and bone loss in femoral head of the IKKe^-/-^ ONFH mice compared with the wild type (IKKe^+/+^) mice (Fig. 5a); moreover, the osteocytes disappeared in IKKe^-/-^ mice with HE staining (Fig. 5b); and the expression of the pro-inflammatory factors Il1b, Il6 and Tnf increased greatly in IKKe^-/-^ mice (Fig. 5c). These data clearly indicated that IKKe knock-out promoted ONFH in methylprednisolone-treated mice and proved the protective effects to bone tissue of IKKe.

## Discussion

In the present study, we found the expression of the inhibitor of IKKe was extremely low in ONFH patients via RNAseq analysis. This finding implied IKKe might have the function of inhibiting osteoclasts, due to the osteoclasts playing a critical role in ONFH. We also proved that the RANKL, an inducer of bone marrow cell to osteoclasts, enables the phosphorylation of IKKe. Studies have described that activation of TBK1/IKKe leads to transcriptional up-regulation of IFN-1 in the innate immune response 14, and activates MAVS and STING to induce IFN-I production and innate immune gene transcription 15. We therefore examined INFα, INFβ and P-IKKe expressions during M-CSF and RANK-induction of bone marrow cells of mice and could not detect any differences in INFα/INFβ during the incubation of RANKL from 0-24 hours, nor were there any differences in P-IKKe in MAVS or STING wild type or knock out mice. Those data implied that the activated IKKe not joined the IFN-1 or viral immune pathways. Further studies need to be done to clarify the mechanism. We tested BAY-985, an inhibitor of IKKa/b and found this inhibitor eliminated the phosphorylation of IKKe, suggesting that IKKa/b may be the kinase of IKKe. To search the possible interaction factors with IKKe, we used the STRING Interaction Network. Interestingly, we found TBK1 interacted with NIK in non-canonical NF-κB pathway. TBK1 was considered to locate upstream NIK and negatively regulate this kinase 16. As TBK1 possesses highly homogeneous characteristics with IKKe, we hypothesized that IKKe may interact with NIK and negatively influence osteoclastogenesis via non-canonical NF-κB pathway. Our experiments proved that IKKe directly interacted with NIK and suppressed its expression. Moreover, we investigated the direct influence of IKKe to osteoclasts. We collected the bone marrow cells from IKKe+/+ and IKKe-/- mice and measured the osteoclast differentiation (TRAP staining). The IKKe knock out mice expressed significantly higher osteoclast intensity in TRAP staining compared with the wild type (IKKe^+/+^), so did the RNA levels of osteoclast related genes, Cstk, Acp5 and Calcr. However, no differences were observed with the expressions of the pro-inflammatory factors Il1b, Il6, Tnf and Nos2 in those two groups. These data proved that IKKe negatively regulates osteoclastogenesis. Finally, we established methylprednisolone-induced ONFH mice model and examined the bone structure and pro-inflammatory factors in IKKe^+/+^ or IKKe^-/-^ mice. As anticipated, IKKe knock-out mice expressed bone structure collapse and osteocytes loss coupled with higher expressions of pro-inflammatory factors Il1b, Il6 and Tnf. Our experiments *in vivo* clearly showed IKKe presents bone tissue-protective effects in methylprednisolone -treated mice.

Previous study has showed that NIK is a key component of the non-canonical NF-κB pathway. NIK triggers ReIB and down-stream signals are essential for osteoclastogenesis and osteoclast differentiation. *In vivo* experiments have proved that knock-out of NIK or ReIB eliminated osteoclastogenesis 17, 18, which suggest that the non-canonical NF-κB signaling cascade is essential for osteoclast proliferation and differentiation 19. IKK-related kinases were first identified as IKKα and IKKβ which drives expression of genes encoding anti-apoptotic factors, cytokines, and other regulatory proteins 20. The later identified TBK1/IKKe possessed the homology sequence as the canonical IKKs so that they were considered as non-canonical factors in NF-κB pathways. IKKe belongs to IκB kinase family and have been linked with adaptive autophagy, immunity, and oncogenesis 10. Researchers have described IKKe as a cancer promotor in breast cancer 21, 22, ovarian cancer and pancreatic cancer 23, 24. The functions of IKKe to osteoclasts have not been comprehensively studied. In our study, we first showed that the IKKe interacted with NIK and down-regulated this component via the non-canonical NF-κB pathway, resulting in suppression of osteoclastogenesis. From the kinase property of IKKe, we hypothesize that other components, such as NIK inhibitors, or NIK polyubiquitination factors are involved in the IKKe/NIK complex. IKKe phosphorylates the unknown inhibitor or degrader of NIK to negatively regulate NIK 25. In addition to the canonical NF-κB pathway regulation, other factor, such as TBK1, was described to interact with IKKe and negatively regulate the NF-κB signaling in a non-canonical way 26. In the nearly further, we believe that more and more factors and pathways will be studied to better understand the mechanisms of IKKe regulation in osteoblasts.

## Conclusion

In conclusion, our study is the first to provide evidence that IKKe interacts with NIK, down-regulates osteoclasts, and possesses protective effect on ONFH.

## Supplementary Material

Supplementary materials.
