# Supplementary material for: IKKe in osteoclast inhibits the progression of methylprednisolone-induced osteonecrosis
Source: Int J Biol Sci. 2021 Mar 30;17(5):1353–60. doi: 10.7150/ijbs.57962 (PMC8040464; doi:10.7150/ijbs.57962)
Supplement: Supplementary file 1 — Supplementary materials. [file ijbsv17p1353s1.pdf]

Supplementary materials

Primers in this study:

Mouse primers:

Cstk sense 5'-ccagtgggagctatggaaga-3'

Cstk antisense 5'-aagtggttcatggccagttc-3';

Acp5 sense 5'-cgtctctgcacagattgcat-3'

Acp5 antisense 5'-gtagtcctccttggctgctg-3';

Calcr sense 5'-cggactttgacacagcagaa-3'

Calcr antisense 5'-gtcaccctctggcagctaag-3';

Il6 sense 5'-ctgatgctggtgacaaccac-3

Il6 antisense 5'-cagacttgccattgcacaac-3';

Tnf sense 5'-CATCTTCTCAAAATTCGAGTGACAA-3'

Tnf antisense 5'- CCAGCTGCTCCTCCACTTG-3';

Il1b sense 5'-aagcctcgtgctgtcggacc-3'

Il1b antisense 5'-tgaggcccaaggccacaggt-3';

Ifnb sense 5'-agctccaagaaaggacgaacat-3'

Ifnb antisense 5'-gccctgtaggtgaggttgatct-3'

Nos sense 5'- GAGACAGGGAAGTCTGAAGCAC-3'

Nos2 antisense 5'- CCAGCAGTAGTTGCTCCTCTTC-3'

Ifna4 sense 5'- GCAATGACCTCCATCAGCAGCT-3'

Ifna4 antisense 5'- GTGGAAGTATGTCCTCACAGCC-3'

Human primers:

IKKe sense 5'- GGCTACAACGAGGAGCAGATTC -3'

IKKe antisense 5'- GGACGCTTGATACTTCTGCACG -3
